# Advanced Photonic Thin Films for Solar Irradiation Tuneability Oriented to Greenhouse Applications

**DOI:** 10.3390/ma14092357

**Published:** 2021-05-01

**Authors:** M. Barragán Sánchez-Lanuza, Amador Menéndez-Velázquez, Antonio Peñas-Sanjuan, Francisco J. Navas-Martos, Isidoro Lillo-Bravo, José-María Delgado-Sánchez

**Affiliations:** 1 Department of Energy Engineering (ETSI), University of Seville, 41092 Seville, Spain; isidorolillo@us.es; 2 Photoactive Materials Research Unit, IDONIAL Technology Center, 33417 Avilés, Spain; amador.menendez@idonial.com; 3 ANDALTEC Technological Center, 23600 Martos, Spain; antonio.penas@andaltec.org (A.P.-S.); francisco-javier.navas@andaltec.org (F.J.N.-M.); 4 Department of Applied Physics (ETSIA), University of Seville, 41013 Seville, Spain; jdelgado17@us.es

**Keywords:** greenhouse, luminescent, FRET, dye, photonic crystal

## Abstract

The world population is growing by 1 billion people every 10 years. There will come a time when there will be more people to feed but less land to grow food. Greenhouses can be the solution to this problem because they provide the highest production yield per m^2^ and also use less water, provide food safety, and offer high quality. Photosynthetic active radiation (PAR) favors vegetable growth with a specific blue and red light ratio. Thus, increasing the amount of red light improves chlorophyll absorption and photosynthetic efficiency. In this article, we present a hybrid system that combines luminescent materials and photonic crystals for better management of the light reaching the greenhouse. The luminescent dyes considered herein are combined ensuring a Förster resonance energy transfer (FRET) nonradiative mechanism to enhance the absorption range. The designed photonic crystal maximizes reflections in the Near-Infrared (NIR) range, and therefore, thermal losses are minimized. Thus, by converting harmful or ineffective radiation for plant growth to the PAR region, we aim to demonstrate growth-condition enhancement for the different vegetables that have been used as a model.

## 1. Introduction

The manufacturing of any product requires energy to transform raw materials into useful goods. The use of renewable energy sources and sustainable conversion processes, aligned with environmental protection, are key parameters for the development of society. Industrial companies have to integrate energy-efficient performance in production management [1]. The human population is expected to exceed 9 billion by 2050, and thus the demand for water, food, and energy will be continuously increasing. Over two-thirds of human water use is dedicated to agriculture. In Asia, the share is four-fifths. Agriculture is also responsible for 13% of greenhouse gas emissions [2]. Therefore, suitable adaptive measures in agriculture are needed to improve its efficiency concerning climate change, water scarcity, and food security.

In the last decades, the idea of using closed greenhouses was assumed in order to save water and energy. Thus, greenhouse agriculture has been considered as an intensive production structure creating aligned boundary conditions of climate control and increasing efficiency of resources [3]. However, greenhouses are one of the most energy-consuming sectors in the agricultural industry. The current tendency is to implement passive control techniques such as natural ventilation instead of forced ventilation, which requires the use of fans [4].

The cost balance of a greenhouse depends on many parameters, but the most important ones are primary energy cost and total production yield [3]. To achieve a successful balance between them, the typical conditions required for a greenhouse are first to guarantee a stable temperature profile, and second to ensure optimum irradiation for energy conversion in the plants, the process known as photosynthesis.

The temperature profile is the most studied parameter in the literature. A greenhouse gets very hot during the day and remains warm enough at night compared with the environmental conditions. To keep temperature distribution constant at the optimum range is essential for optimal crop growth [5,6,7,8,9]. Transitory or constant high temperatures cause a range of morpho-anatomical, physiological, and biochemical changes in plants. They affect plant growth and development and might engender drastic reduction in their economic yield [10]. This result has as consequence in active and passive thermal management techniques [11,12].

The second parameter, external irradiation, is critical because it influences the photosynthesis crop process, and also the temperature distribution inside the greenhouse. Photon energy of the irradiation spectrum is absorbed by chlorophyll molecules in chloroplasts, where the solar energy is converted into chemical energy. Hemming et al. [13] reported that photosynthetically active radiation (PAR) is typically in the range of 400 to 700 nm, and the shape of PAR is almost universal: small variations are due to development phase, place of growth, water supply, etc. This is due to the fact that all plants contain the same photochemical system based on the same pigments that govern the leaf spectral absorption. There are two types of chlorophyll pigments, known as A and B; the first one is responsible for blue (430 nm) and red (662 nm) light absorption, while chlorophyll type B uses a similar range with absorption peaks at 453 and 642 nm approx. Other secondary pigments, like beta-carotene, exhibit small absorption peaks in the range of 400 to 500 nm [14]. However, green and far-red irradiation is not absorbed.

Incoming irradiation out of this spectral range only generates undesired effects that reduce the greenhouse performance: near ultraviolet (NUV) radiation affects film degradation, plant damage, and pollinator behavior, and near-infrared (NIR) radiation is a direct source of heat. Much of NIR radiation penetrates into greenhouses and causes heat loads [15].

There are some previous research studies analyzing the effect of using photoselective plastic films to modify the light spectrum that enters the greenhouse, so the photosynthesis of the plants can be affected. Fletcher et al. [16] demonstrated that marketable yield per plant was 51% greater using films with different red/far-red transmission ratios. Wilson and Rajapakse [17] tested plant response to photoselective plastic films with varying spectral transmission properties. Van Haeringen et al. [18] studied phthalocyanine derivatives that were prepared and incorporated into polymer films in order to be used as spectral filters for the modification of plant growth.

In sum, two phenomena have to be considered to improve greenhouse performance: (1) avoid transmission of NIR inside of the greenhouse in order to minimize overheating during the day and cooling during the night; and (2) convert green light into red light to enhance the chemical energy transformed by chlorophylls. Both objectives can be achieved with the combination of photonic crystals and luminescent materials, as thin-film coatings or dye-doped polymers on the greenhouse structure (either glass or polymer-based material).

Photonic crystals (PCs) are periodic dielectric structures that are designed to form the energy band structure for photons, and either allow or forbid the propagation of electromagnetic waves of certain frequency ranges, making them ideal for light-harvesting applications [19]. Photonic crystals are structured materials also present in nature: for example, in ants, they reflect light and therefore reduce the ants’ temperature. The easiest way to design a PC is to create a structure of materials with a well-defined periodic patterning in the dielectric function so that, depending on the number of layers, their thicknesses and refractive indexes, transparency, and reflectivity can be tuned. Therefore, PCs can act as spectral selective mirrors for certain wavelengths by selecting the materials properly.

Thus, the appropriate combination of the PC concept for greenhouse energy-management purposes might be suitable to remove heat radiation (infrared radiation) from the walls, and then to minimize internal temperatures during the day. Moreover, this wavelength does not affect the crop-development mechanism: 1000 nm is the critical wavelength; up to this value radiation is transmitted, and it is reflected onward.

Additionally, photoluminescent materials absorb light at a certain wavelength, and the light is re-emitted at another wavelength with lower energy. This means that they are able to tune the incoming solar spectrum; for example, by transforming the green spectral range to the red part of the spectrum. There is an available wide portfolio of organic luminescent molecules, so downshifting the described process is easy to adjust to different applications, such as sensors [20], energy [21], lasers [22], and greenhouses. The operation of this luminescent layer is based on the following principle: one or more dyes are embedded in a host matrix, a highly transparent medium with a high refractive index. Photons that enter the host material are absorbed by the luminescent dyes and re-emitted in random directions. It is noted that dye selection and its concentration could be optimized in order to match the spectral sensitivity for the photosynthesis of the plants.

So, luminescent materials can be directly applied on greenhouses to improve the useful energy requested for crop development (PAR), or even Luminescent Solar Concentratos (LSC) can be integrated into agricultural structures to benefit the photosynthesis processes at the same time that electricity is produced, either for self-energy consumption or for selling energy to the electrical market. The scope is to seize the opportunity that PAR is not influenced by green solar spectrum, so thanks to this family of materials, this energy can be easily transformed into the red part of the spectrum for a better crop exploitation.

During the 1990s, Novoplansky et al. [23] analyzed the effect of incorporating organic luminescent molecules in greenhouse polymer covers. They observed how solar irradiation was tuned to optimize the PAR effect on greenhouse crops to improve the vegetable lifetime cycle. It was reported that the luminescent molecules provided, as their main benefit, the reduction of the green light, which in turn reduced stress on the crop, resulting in lower leaf temperatures, greater disease resistance, and high crop yields.

Gonzalez et al. [24] investigated the efficiency of new fluorescent polymer films, especially those including additives which work as green to red light converters. Hemming et al. [25] investigated the effect of new developed fluorescent greenhouse films on the growth of *Fragaria × ananassa* “Elsanta”. In order to optimize light quality and quantity growth, several photoselective greenhouse-covering materials were developed, with most of them based on red dyes (with photoluminescence between 610 and 690 nm) in different concentrations.

Corrado et al. [26] investigated the hybridization of photovoltaic energy generated by a luminescent solar concentrator and the greenhouse impact. Solar irradiation was absorbed by the luminescent dyes embedded in a polymethyl methacrylate (PMMA) rigid sheet, and part of the re-emitted light was guided to solar cells, while the rest was transmitted to the plants inside of the greenhouse. The intention was to analyze the double benefit of the luminescent films: to adapt the light to the optimum PAR, and to generate electricity for the self-consumption of the installation.

Simakin et al. [27] and Wang et al. [28] explored alternative materials to ensure the same goal. Simakin improved the light transmitted by the polymer when it was doped with rare earth compounds, while Wang used, for the same objective, quantum dots based on Cd_(1−x)_Zn_x_Se, but they concluded that their film had a low quantum yield, so another chemical compound approach was needed.

The majority of these experiments considered the commercial luminescent material from BASF, called Lumogen 305 Red. However, the main disadvantages observed in this type of organic dye were: (1) narrow absorption spectrum; (2) high reabsorption due to the overlapping of the absorption and emission peaks (Stokes losses); and (3) limited tuneability to provide this service for a wider range of PAR specifications.

An interesting alternative strategy is to consider a mix of dyes embedded in the host material, ensuring they transfer the energy between them in a nonradiative mechanism. This is called Förster resonance energy transfer (FRET) [29,30]. It has been proved with good results in other scientific areas, such as biology [31], physics [32], and materials science [33]. So, thanks to this phenomenon, the proper selection of the dyes resulted in a thin-film optical window with color tuneability, better luminescence properties, and a wide absorption spectrum.

The aim of this research study is to provide a new novel design based on the combination of photonic crystals and multiple luminescent dyes transferring energy between them with a FRET nonradiative mechanism. Thanks to this original approach, the flexible film will ensure: (1) reflection of the light in the infrared spectrum, responsible for increasing the internal temperature of the greenhouse; (2) wide absorption spectra range for those wavelengths that are not critical for photosynthesis; (3) light photoemitted in the proper energy compared to PAR; (4) minimization of Stokes losses; and (5) a high degree of tuneability of the system, upon the dyes selected, to modify the absorption and emission peaks, upon the desired PAR and the boundary conditions for each specific location.

## 2. Materials and Methods

Luminescent films were manufactured with a combination of three dyes to cover the green absorption spectrum range from 450 to 600 nm c.a., and to improve photoemission around 650 nm c.a., according to PAR crops. The selected dyes were Lumogen Red (LR305), 4-(Dicyanomethylene)-2-methyl-6-(4-dimethylaminostyryl)-4H-pyran (DCM) and 2-[4-[4-(dimethylamino)phenyl]-1,3-butadienyl]-1,3,3-trimethyl-3H-indolium perchlorate (LDS), which were all used as received; the first one from BASF and the others from Exciton. The host material selected was a low-density polyethylene (LDPE) from LyondelBasell, as it is currently the most commonly used material for the manufacturing of conventional greenhouse films.

**Figure 1:**
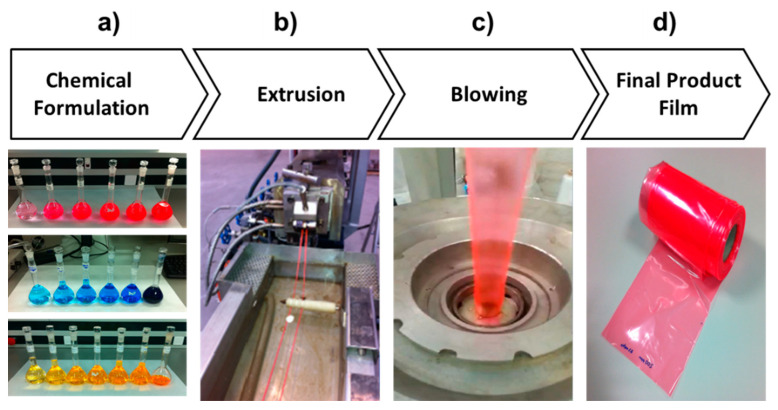
Manufacturing process: (**a**) chemical solutions with different dye concentrations; (**b**) extrusion machine with the luminescent material embedded in the LDPE matrix; (**c**) blowing machine; and (**d**) polymer substrate coil based on LDPE doped with luminescent dye.

This research study comprised two stages: laboratory and pilot scale (Figure 1). The samples processed during the lab-scale stage were prepared using a spin-coating (Laurell, WS-650Mz-8NPP). Luminescent solutions were prepared from a mixture of the as-received powered dyes, polymethyl methacrylate (PMMA) host material, and chloroform (Sigma Aldrich). A total of 300 mg of PMMA and 4 mL of chloroform were stirred during 48 h to ensure a homogeneous solution. Then, the dyes were added to the PMMA solution, resulting a 1% w/w mixture, the optimal concentration to avoid quenching. Finally, the solvent was evaporated on a hot plate at 50 °C.

Compounding at pilot scale was carried out at 140 °C by gradually adding LDPE and the luminescent dye to a solution of p-Xilen (Sigma Aldrich). The solution remained for 2 h under stirring conditions to ensure the optimal mixing of the LDPE and dye molecules. After this time, p-Xileno was removed from the mixture by means of a distillation procedure to obtain a homogeneous solid. Different dye concentrations were tested from 0 to 5% w/w. One masterbatch of luminescent dye and LDPE was prepared from the previous solid and raw LDPE by employing a corotating twin-screw extrusion line with a pelletizing system (SIEPLA model SHJ-20). All resulting samples were processed in a mill machine with stainless steel blades (J Purchades model 250) in order to ensure homogeneity with an average particle size of 3 μm. The extrusion process was carried out by using a specific thermal profile of 90 °C/150 °C/180 °C/190 °C. During the entire process, the screws were rotating at 300 rpm. Finally, three different regrinds for each luminescent combination were fabricated: 100, 200, and 300 ppm. To end the procedure, luminescent film was obtained (Figure 1) from a blown film extrusion machine (Sincerity Plastic Machinery, model SJ50-700) with 250 mm width and 10 μm thickness, by using a thermal profile of 150 °C/180 °C/195 °C.

**Figure 2:**
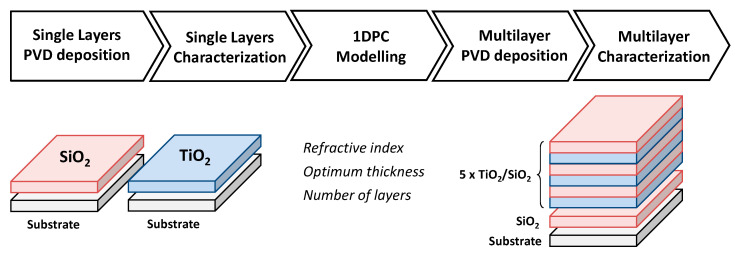
1DPC manufacturing process: magnetron sputtering deposition (PVD) of single SiO_2_ and TiO_2_ layers, and their characterization; modeling to find the optimum parameters (thicknesses, sequence, and number of layers stacked); and finally, multilayer magnetron sputtering deposition.

One-dimensional photonic crystal (1DPC) structures were designed to reflect the maximum energy at the near-infrared spectral range. For that purpose, periodic dielectric structure was defined as 13 layers alternating SiO_2_ (1) and TiO_2_ (2), resulting in the sequence 1 2 1 2 1 2 1 2 1 2 1 2 1 (Figure 2). Refractive index and thickness for SiO_2_ were n_1_ = 1.3 and d_1_ = 229 nm, respectively. and the values selected for TiO_2_ were n_2_ = 2.3 and d_2_ = 130 nm.

Dielectric layers were deposited by DC magnetron sputtering (44 Solar) using ceramic targets (99.999%) with a dimension of 500 × 100 mm^2^. Low-density polyethylene film was used as a substrate, with a dimension of 300 × 300 mm^2^. To avoid any contamination from the substrate, it was cleaned using a glow discharge in the load-lock chamber. The process chamber was evacuated below 5 × 10^−7^ mbar prior to any deposition, and all depositions were carried out in an Ar/O_2_ mixture under constant pressure of 2 × 10^−3^ mbar. The O_2_ flow rate was selected in order to operate beyond the transition region of the cathode voltage versus oxygen flow curve, where the deposition fully works in the oxide mode. The process was performed at plasma temperature.

Thickness was measured using a mechanical profilometer (KLA Tencor model P-6,). Optical properties were characterized using a UV-VIS spectrometer setup including a Xe-lamp (Newport model 71228), monochromator (Oriel Cornerstone 260), integer sphere (Labsphere 3P-GPS-030-SF), and UV-VIS detector (Newport 71610). The signal was recorded using a dual lock-in amplifier (Stanford Research SRS-830).

## 3. Results and Discussion

The modification of light for plant growth was based on the principle that not all energies are equal, with red wavelengths having been proven as the most photosynthetically efficient energies. By converting a fraction of the incoming blue light and green light to red light, this film installed in the greenhouse increased the photosynthetic efficiency of light reaching the canopy, allowing growers to favor root development and flowering. These principles were demonstrated in this research study.

### 3.1. Luminescent Characterization

**Figure 3:**
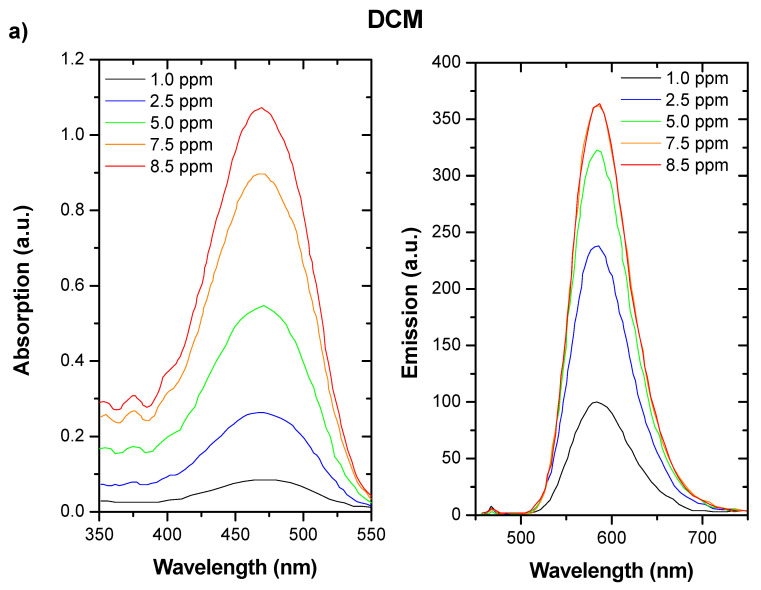

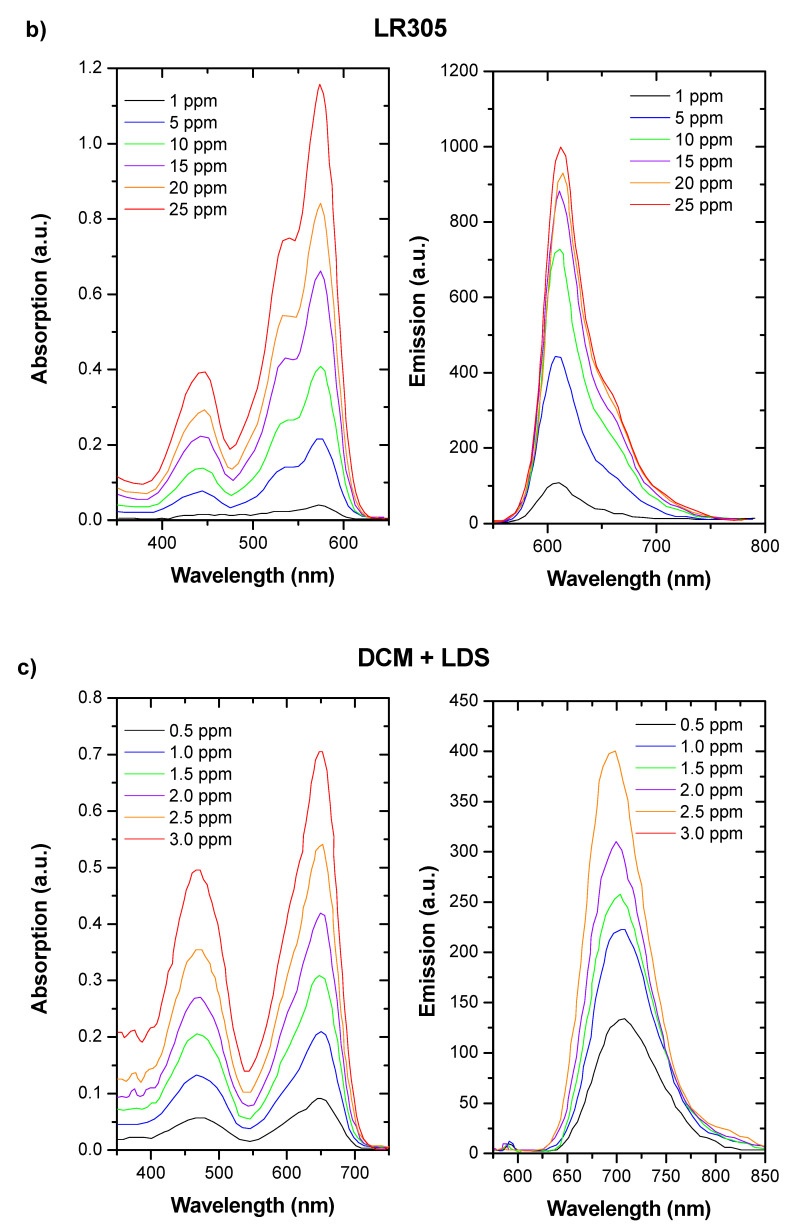

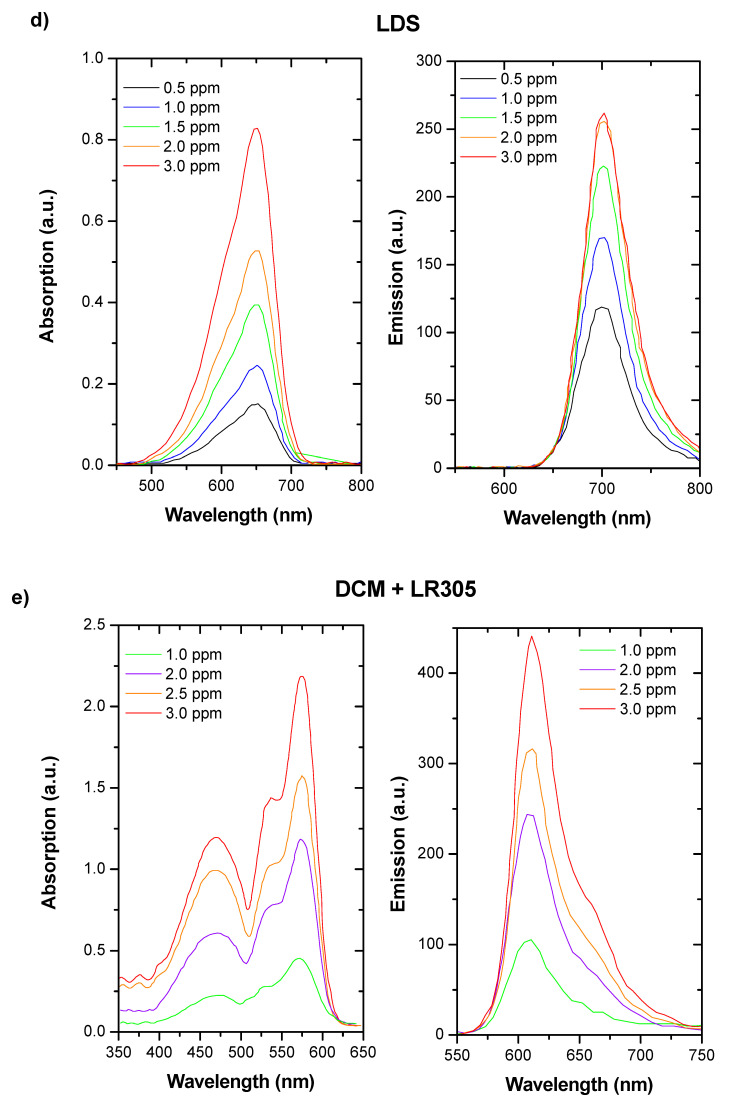
Absorption and emission spectra obtained for different dyes: (**a**) DCM, (**b**) LR305, (**c**) LDS, (**d**) DCM + LDS, and (**e**) DCM + LR305. Emission peaks were aligned with PAR requirements, and combination of DCM + LDS offered the advantage of wider absorption spectra.

Figure 3 shows the absorption and photoemission spectra for the prepared luminescent films, at different concentration levels. The dyes were dissolved in CHCl_3_ at laboratory scale in order to identify whether their photoemission peaks were aligned with the PAR spectrum requested. It was observed that all dyes had a maximum luminescent emission in the range of approximately 600 nm (DCM and LR305 (Figure 3a,b) and 700 nm (LDS, Figure 3d)). Moreover, the absorption of these dyes was located in the green range of the solar spectrum, which is the best condition according to the PAR crop’s response. To improve the absorption spectra range, DCM was mixed with LDS or LR305 in the same solution, ensuring FRET conditions (Figure 3c): the acceptor dye (DCM) was excited and emitted energy was transferred to the donor dye (LDS or LR305) in a nonradiative mechanism. Emission from DCM + LDS and DCM + LR305 was obtained by exciting the system with a monochromatic source at 468 nm, corresponding to the acceptor dye absorption spectra. Under this condition, the emission obtained corresponded to the LDS dye without being directly excited, demonstrating the efficiency of the FRET process.

It is remarkable that the absorption spectra were dramatically increased by increasing the concentration of the dyes used, and the absorption range was enhanced when DCM was combined with LDS or LR305. This increase was accompanied by a rich vibrational profile as the spectra became broader around the main absorption peaks. This behavior can be explained by the formation of dimers and higher molecular weight aggregates recognized by large spectral broadening, and obvious deviation from the Lambert–Beer law. Two main types of molecular aggregates can be created by high concentrations of fluorescent dye molecules: J-aggregates, which are face-to-face molecular arrangements that cause a bathochromic (red) shift; and H-aggregates, which are face-to-face molecular arrangements that yield a hypochromic (blue) shift [34].

**Figure 4:**
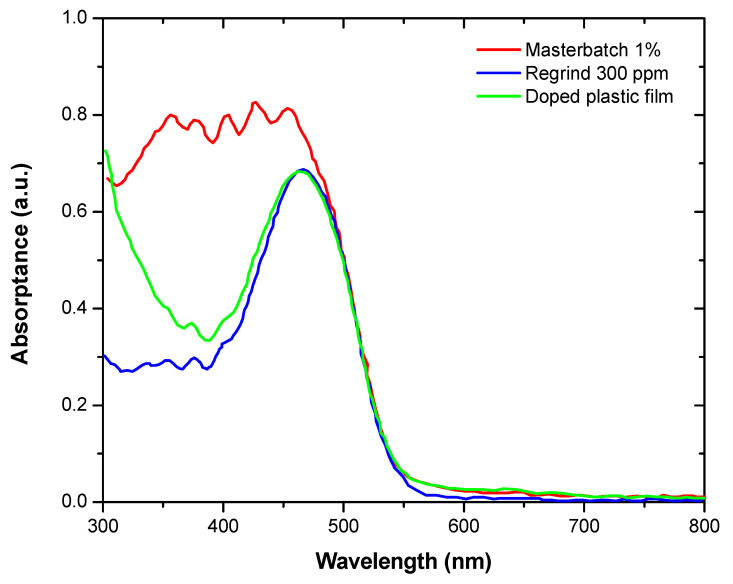
Absorption characterization at different manufacturing stages of the luminescent film. It is shown how the LDS absorption band was degraded after the second extrusion step.

These lab designs were scaled up to a production scale to obtain large LDPE films doped with luminescent dyes, following the procedure described in Section 2. However, it was observed that the DCM + LDS film failed in terms of luminescent properties, so a reverse-engineering process was carried out to check if the doping additives revealed the same optical properties before and after the film-manufacturing process. Figure 4 shows that the DCM + LDS proposal was degraded during the extrusion process: comparing the absorption spectra of these samples, it was observed that the optical response assigned to the LDS dye fully disappeared in the blown film; the DCM dye remained in good condition with slight modification at a lower wavelength, which is explained by optical interferences with the LDS residues originated during the degradation process. Thus, the best samples selected for the pilot test were DCM and DCM + LR305, based on the FRET concept. Both were compared against conventional LR305, which has been previously used by other authors [22,23,24,25], and none of them experienced any degradation phenomena during the industrial manufacturing. Nevertheless, the approach of using a combination of two dyes embedded in the LDPE host material had the advantage of providing a higher absorption level than the conventional LR305 single dye.

**Figure 5:**
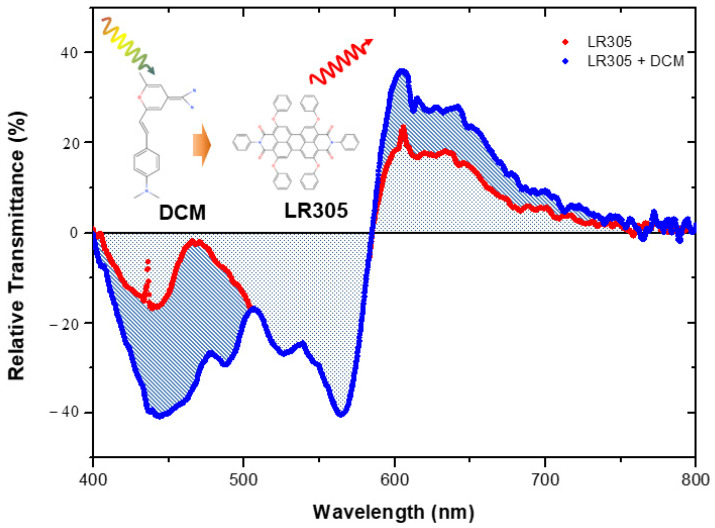
Normalized transmittance of the LDPE films doped with LR305 (red) and FRET system based on LR305 and DCM (blue).

The effect of the different dyes at lab scale was investigated at pilot scale. Industrial LDPE doped films were manufactured, and their normalized transmittance spectra to the reference sample (LDPE undoped) are illustrated in Figure 5. Transmittance of the doped LDPE films, compared to the reference undoped LDPE film, showed two regions. In the high-energy range (from 400 to 570 nm), it was observed that the dyes reduced the transmittance compared to the undoped LDPE film (negative values). This was as a consequence of the absorption of the DCM and LR305 molecules. The area defined between the reference horizontal axis and the red dots was proportional to the energy absorbed by the film. Theoretically, this energy is photoemitted (relative transmittance positive) by the acceptor dye (LR305), which is quantified in the area delineated between the red dots and the reference horizontal axis in the low energy region (from 570 to 750 nm). The experimental difference found in these values was understood as thermal losses exciting phonons in the doped film’s atomic structure.

Absorption was significantly increased when the LR305 molecule was combined with the DCM one (blue dots) with respect to single LR305 dye doping (red dots). Therefore, photoemission intensity increased, but the spectral profile remained the same, as it was expected in the FRET nonradiative process. When both dyes were mixed, two major absorption bands were observed; the first one was located in the UV region around characterizing the electronic transition of the first excited singlet state (S_0_ → S_1_) of the DCM molecule. The second band lay in the green region, corresponding to the electronic transitions of the LR305 perylene molecule. The absorption contribution from LR305 remained, but the DCM extended the absorption spectra to a high-energy region. Both dyes, DCM and LR305, were transferring energy in a nonradiative mechanism (FRET); this phenomenon explains why the fluorescence spectra were similar to the single LR305 dye, but with the additional benefit of amplifying the intensity of the light photoemitted, in good agreement with the previous results achieved at the laboratory scale. Moreover, this photoemitted light was well aligned with PAR requirements to improve the photosynthesis process.

Table 1 shows the transparency of the films calculated considering the European Standard EN 410:2011 (determination of luminous and solar characteristics of glazing), and the optical properties measured.

### 3.2. Photonic Crystal Characterization

Photonic crystals are periodic dielectric structures that are designed to form the energy band structure for photons, which either allows or forbids the propagation of electromagnetic waves of certain frequency ranges, making them ideal for light-harvesting applications [35], such as avoiding the overheating of greenhouses. In our prototype, based on a 60 µm thick luminescent layer of a doped LDPE matrix, we analyzed the effect of integrating a dielectric mirror, also known as a distributed Bragg reflector or 1DPC. The periodic structure alternated silica (SiO_2_) and titania (TiO_2_) layers.

In this work, a multilayer structure consisted of a periodic structure of SiO_2_ (layer 1) and TiO_2_ (layer 2) according to this pattern: 1 2 1 2 1 2 1 2 1 2 1 2 1. The thickness of the first layer (SiO_2_, low refractive index) was optimized to 229 nm, while the thickness of the second layer (TiO_2_, high refractive index) was selected to be 130 nm. These refractive indices and the thickness of the layers were very large, and directly controlled the strip space of the photonic crystal. These parameters were used to model the reflectance, and the Bragg peak obtained was limited from 1000 to 1600 nm, so the NIR region was not transmitted inside of the greenhouse. Furthermore, this optical response showed that there was not any optical incompatibility between the luminescent material and the photonic crystal, but both of them were well coupled to ensure maximum performance of the films.

**Figure 6:**
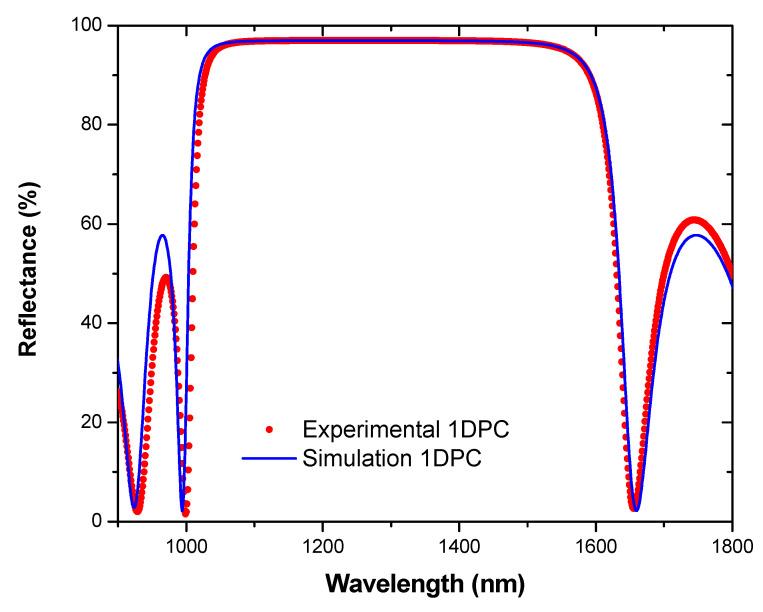
Bragg peak characteristics for the 1DPC SiO_2_/TiO_2_ designed for reflecting the NIR solar spectrum. Red dots correspond to the experimental reflectivity characterization, and the blue line is the simulated optical response.

The simulation and experimental characterization are given in Figure 6. The experimentally measured reflection of the 1DPC showed a good agreement with the theoretical reflection spectra simulated to design the pattern of SiO_2_/TiO_2_. The measured high reflection bands exactly matched with the simulated high-reflection bands. The interface fringes outside of the high-reflection bands almost matched with the theoretically generated fringes. We observed that most of the power irradiation was reflected from 1000 nm, which corresponded to the NIR region. The Bragg peak was centered at approximately λ_0_ = 1300 nm. However, the parameter λ_L_ represented the opening wavelength of the photonic band, and the parameter λ_H_ the wavelength of the closing. ∆λ = 600 nm was the photonic bandwidth, which represented a wider spectra range to reflect the low-energy photons corresponding to the NIR region, minimizing the internal temperature of the greenhouse. The index contrast between layers with high and low refractive index had a significant influence on the Bragg peak’s characteristics: central position and width. For instance, another theoretical model, replacing the TiO_2_ layer with Si_3_N_4_ (120 nm), and using the same periodic patterns (N = 13), offered a wider Bragg peak (900 nm) centered at 1450 nm.

The above results indicate that the SiO_2_/TiO_2_ 1DPC manufactured had infrared spectrally selective high reflectivity, thus meeting the requirements for our greenhouse design. In addition, the dielectric materials covered the entire 1000–1600 nm spectral band. This range of energy reflected might be increased by replacing SiO_2_ with Si_3_N_4_ and keeping the same pattern. The achieved high-reflection band was much wider than the values reported by Chiasera [36], Scotognella [37], and Jena [38], in the NIR region for a TiO_2_/SiO_2_ periodic photonic crystal.

### 3.3. Crop Growth Influence

A side-by-side comparison was conducted using two identical research greenhouses, one covered with the doped luminescent and photonic crystals using LDPE host materials, and the other one with the conventional LDPE films. To investigate the impact of the solar spectrum modification, the following indicators were analyzed: (a) tomato canopy performance (productivity and physiology), and (b) greenhouse internal microclimate (uniformity and crop-microclimate interaction). In addition, a survey study was conducted in the same greenhouses to find the performance of lettuce, herbs, and tomato seedlings.

Three cropping zones were established in each greenhouse: (1) tomato crop production zone with Rockwool-based growing media and a high-wire system (with two cultivars, “Campari” and “Tymoty”); (2) lettuce (Rex and Magenta varieties) production zone with a deep-flow hydroponics system; and (3) an ebb-and-flow bench system to produce seedlings and herbs on a bench as part of the survey study.

The following observations were obtained after one week of monitoring (Table 2): compared with the plants in the control greenhouse, tomato plants under optically modified LDPE film exhibited a faster growth rate (for example, extension of stem and leaves).

Two lettuce varieties, Rex and Magenta, were grown; the lettuce was seeded, and seedlings were transplanted one week later. A modified Hoagland nutrient solution was used, and pH was maintained at 6.64 during the production period. The results obtained in the first crop cycle are shown in Table 3: (a) Lettuce plant growth (increase in size) was faster under the prototype film than under control greenhouse; (b) the enhanced leaf expansion increased overall photosynthetic capacity, and thereby the growth; and (c) growth data obtained at younger seedling stages showed a more pronounced effect of glazing, suggesting the plant growth enhancement under luminescent prototype film was more associated with the enhanced leaf expansion.

**Figure 7:**
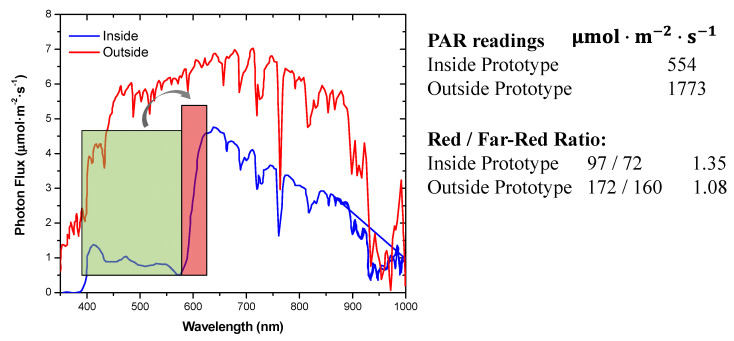
Light quality under the prototype greenhouse, using luminescent-doped LDPE films coupled to 1DPC film.

The light quality was measured using a spectroradiometer on a clear sunny day, after the prototype installation. The light quality is shown in Figure 7. The data show that the prototype cut a significant portion of the UV radiation starting at 370 nm, and also cut the light in the green range of the solar spectrum, with enhanced light quality at 600–650 nm with red light. These data, added to the previous optical characterization of the film, suggest that the luminescent prototype had about 30% light transmission based on the daily light integral (DLI) and in the PAR range. It was also observed that the diffuse component of the light was higher in the prototype greenhouse compared to the control one. The diffuse property of the luminescent film will be further investigated with future measurements using diffuse and direct horizontal solar radiation sensors in both type of greenhouses.

## 4. Conclusions

This research study demonstrated that the combination of an LDPE film doped with luminescent dyes and a photonic crystal improved the greenhouse’s performance. The radiation transmitted by the film was modified to match the PAR response of the crops: green light was re-emitted into red light, and the NIR spectra responsible for the thermal losses was minimized.

The right combination of two dyes (DCM and LR305) to ensure a FRET nonradiative mechanism resulted in a system with a wider absorption range when compared with results reported previously by other authors (LR305). The wider the absorption spectrum was, the higher the photoemission measured, with obvious better optical performance. The 1DPC design showed a reflectivity over 97% in the NIR spectrum (from 1020 to 1680 nm), with excellent matching between theoretical and experimental values. Thus, thermal conditions inside of the greenhouse remained isolated, as the low-energy photons could not be transmitted by the film.

In addition, it was demonstrated that the optical properties of the film (luminescent dyes and the photonic crystal) could be easily modified to adapt the greenhouse materials to any specific location (solar irradiation, environmental temperature, etc). Additional dyes could be added to increase or modify the absorption and emission spectra. Moreover, the TiO_2_ and SiO_2_ thicknesses and refractive indexes of the 1DPC could be tuned to adapt the film’s reflectivity to a different spectrum range.

Two vegetables were selected to evaluate the benefit of this greenhouse film: lettuce and tomato crops. Crop growth under these conditions exhibited faster development of leaves and extension of stems (elongation). The enhanced leaf expansion increased overall photosynthetic capacity, and thereby the growth.

The technology described in this paper can manipulate sunlight in a similar way to LEDs, without any use of electricity at all. Future work will concern deeper analysis of this design in different climate locations; an exhaustive energy model will be elaborated, and the photostability of the films over long-term periods will be evaluated. This information will be checked by the market to determine a better economic and feasibility model.

## Figures and Tables

**Table 1:** Optical properties of the films manufactured, using different doping molecules: DCM, LR305, and DCM + LR305.

Optical Properties	DCM	LR305	DCM + LR305
Transparency (%)	94.2	99.5	94.5
Absorption range (nm)	400–490	470–580	400–580
Max. emission (nm)	580	610	610

**Table 2:** Plant stem length and number of leaves monitored for one week.

Cultivar	Greenhouse	Steam Length (cm)	Number of Leaves
Tymoty	Control	196.6 ± 8.4	15.2 ± 0.8
	Prototype	223.0 ± 12.2	15.6 ± 0.5
Campari	Control	159.3 ± 11.5	15.0 ± 0.8
	Prototype	161.8 ± 16.3	16.2 ± 1.5

**Table 3:** Lettuce fresh and dry weight and leaf area comparison.

Cultivar	Greenhouse	Head Fresh Weight (g)	Root Fresh Weight (g)	Root Dry Weight (g)	Leaf Area (cm^2^)	Shoot Fresh Weight (g)	Shoot Dry Weight (g)
Rex	Control	63.5	3.2	0.25	46.5	1.3	0.10
	Prototype	71.8	2.6	0.10	88.7	2.5	0.15
Magenta	Control	66.1	3.4	0.14	43.6	1.3	0.08
	Prototype	79.2	3.1	0.11	74.3	2.3	0.10

## Data Availability

Data available upon request.
